# Progress in Neurology

**Published:** 1903-06-13

**Authors:** 


### Progress in Neurology.

(*Concluded from page* 169.)

**Raynaud's Disease.**—Cushing[17] has employed a novel mode of treatment in a case of this disease affecting a woman, aged 35 years, in whom a condition of local asphyxia of all the digits had been present for several months with exacerbations of almost daily occurrence. On several occasions these attacks were so severe that slight superficial patches of terminal gangrene had affected the pads on one or more of the toes or fingers. Morphia had been the only drug which gave relief. Cushing's treatment consisted in the application of a tourniquet, for one or two minutes' duration, round the upper arm or thigh. On its removal a bright flush of the extremity followed, with increase of surface temperature, and much relief from the pain. This procedure was carried out regularly every day, with the result that in six weeks the patient had quite recovered and her extremities had resumed their original physiological tint. The treatment probably depends upon the physiological "blocking" effect of the elastic constriction on the peripheral vaso-motor nerves. The temporary interruption of function allows the terminal arterioles to relax, and a state of hyperæmia to take the place of the local asphyxia which has resulted from the pre-existing spasm. The repetition of the process appears to bring about a cure by the local vascular conditions in the extremities becoming so altered by the periods of active circulation that slight peripheral stimuli no longer produce the intense reflex constrictor responses.

**Myopathy.**—Batten[18] contributes an account of the various types of myopathy, which have the common features of a slow progressive loss of power in muscles or groups of muscles, associated with atrophy or hypertrophy, absence of fibrillary contraction, and absence of any qualitative electrical change. The atrophied muscles do not in their grouping correspond to any affection of the spinal cord or nerve; there is a marked tendency for the disease to be hereditary and to affect various members of a family. The muscles of the eyes, larynx, and pharynx, and the diaphragm, are never affected. The following groups can be distinguished :—(1) The infantile type; in which the disease begins in infancy, the baby does not learn to sit up at the usual age, and even when this is learnt the child has considerable difficulty in maintaining the erect position. They walk very late or not at all. They are usually very intelligent and contented. All the muscles are very small, but there is no movement which cannot be performed, and there is no localised atrophy or hypertrophy of any muscle. Sensation is normal and the superficial reflexes present. The knee-jerks are diminished or absent according to the degree to which the atrophy has progressed. The reaction to faradism and galvanism is much diminished, and they are very insensitive to the pain of a strong faradic current, though normally sensitive to galvanism and to ordinary forms of stimulus. The explanation of this curious phenomenon is obscure. (2) In the facio-scapulo-humeral or Landouzy-Dejerine type, the disease may commence either in early childhood or in adult life; it is markedly hereditary and may be transmitted by the male or female to the male or female with like frequency. The face is almost expressionless, with a wide, generally open, mouth. The eyes cannot be fully closed, the brow cannot be raised, the cheeks cannot be blown out and the patient cannot whistle. The movements of the eyes, tongue, masseters, and palate are normal. There is marked wasting about the shoulders and upper arm muscles, and about the gluteal and thigh muscles, whilst below the elbows and knees the muscles are well-developed. The sphincters are unaffected. (3) The juvenile type of Erb or scapulo-humeral type is by some regarded as a variety of the last-mentioned type, but differs in that the face is unaffected, hypertrophy is occasionally present, and it commences in the second decade of life, not from birth. The disease affects families and both sexes alike. (4) The pseudo-hypertrophic type, unlike the others, affects the male sex more frequently than the female, and the disease is usually transmitted through a healthy mother who has brothers affected. The features are too well known to be recapitulated. (5) The peroneal type is markedly hereditary, the disease passing from father or mother to daughter or son indiscriminately, even through five generations. It is usually manifest before ten years of age. The atrophy begins in the peroneal muscles of the legs, and affects the more distal portions of the muscles before those more proximately situated. There are marked vaso-motor disturbances, and some alteration of sensation is often present. The disease is of slow progress, and after the legs have become affected the small muscles of the hands begin to suffer. It is to be remembered that intermediate between the above-mentioned five types of myopathy, cases occur which cannot be classified as belonging exclusively to any one type.

[17] Jour. of Nerv. and Ment. Dis., Nov. 1902. [18] Clin. Jour., Jan. 14, 1903.

